# Single arterial access technique for simultaneous dual mechanical circulatory support in cardiogenic shock

**DOI:** 10.1186/s13019-024-02581-6

**Published:** 2024-02-03

**Authors:** Alexander Fardman, Nikolay Dranishnikov, Gaik Nersesian, Volkmar Falk, Evgenij Potapov

**Affiliations:** 1 Cardiology Division, Leviev Cardiothoracic and Vascular Center, Sheba Medical Center, Tel HaShomer, Israel Affiliated to Sackler School of Medicine at Tel-Aviv University, Ramat-Aviv, Israel; 2 Department of Cardiac Anesthesiology and Intensive Care Medicine, Deutsches Herzzentrum der Charité (DHZC), Augustenburger Platz 1, 13353 Berlin, Germany; 3 Department of Cardiothoracic and Vascular Surgery, Deutsches Herzzentrum der Charité (DHZC), Augustenburger Platz 1, 13353 Berlin, Germany; 4 DHZK (German Centre for Cardiovascular Research), Partner Site Berlin, Berlin, Germany; 5 Department of Cardiothoracic Surgery, Charité – Universitätsmedizin Berlin, corporate member of Freie Universität Berlin, Humboldt-Universität Zu Berlin, and Berlin Institute of Health, Berlin, Germany; 6 Department of Health Sciences and Technology, ETH Zürich, Zurich, Switzerland

**Keywords:** Cardiogenic shock, Intra**-**aortic balloon pump, Veno-arterial extracorporeal life support, Ventricular unloading

## Abstract

Temporary mechanical circulatory support is a treatment of choice for patients in severe cardiogenic shock. Combining veno-arterial extracorporeal life support (ECLS) with devices that enable left ventricular unloading emerges as a promising strategy to diminish detrimental effect of elevated left ventricular afterload and to improve survival. However, the need to establish multiple arterial access sites remains a major drawback of this approach due to a significant rate of vascular complications. We describe herein a case of a single arterial access for ECLS and intra-aortic balloon pump using axillary artery that may provide a simple, modular and flexible approach for escalation or de-escalation of mechanical circulatory support.

**Supplementary Information:**

The online version contains supplementary material available at 10.1186/s13019-024-02581-6.

## Background

Temporary mechanical circulatory support (tMCS) is recommended for patients with cardiogenic shock unresponsive to medical therapy [1]. Left ventricular unloading by combining veno-arterial extracorporeal life support (va-ECLS) with either an intra-aortic balloon pump (IABP) or a percutaneous microaxial left ventricular assist device (Impella 5.5, Abiomed, Danvers, MA, USA) could decrease left ventricular afterload and myocardial oxygen consumption, facilitate ventricular recovery, and improve survival [2]. However, this approach increases the rate of vascular complications due to the use of two separate arterial access sites [2]. While insertion of va-ECLS and Impella using a single arterial site technique (ECMELLA) was previously described [3], simultaneous implantation of IABP and va-ECLS usually requires two separate arterial accesses. We describe the first case of simultaneous insertion of IABP and va-ECLS using a single arterial site.

## Case presentation

A 56-year-old lady was admitted to a different hospital with late arrival anterior ST-segment elevation myocardial infarction (STEMI). Her prior medical history was significant for hypertension and obesity, without prior coronary artery disease. Coronary angiogram revealed acute total occlusion of the left anterior descending artery requiring percutaneous coronary intervention with the deployment of two drug-eluting stents. Despite reperfusion, the patient developed acute cardiogenic shock, and transthoracic echocardiography (TTE) revealed a left ventricular ejection fraction (LVEF) of 15%. Despite infusion of inotropes and vasopressors, the patient continued to deteriorate, developed pulmonary edema and was intubated. Despite further medical treatment, the patient’s condition deteriorated even further, the lactate level rose to 7.9 mmol/L and the urine output decreased (Society for Cardiovascular Angiography and Interventions [SCAI] classification stage D). At this point the decision was made to transfer the patient to our institution for tMCS initiation.

Upon arrival the patient was supported with adrenaline 0.9 mcg/kg/min, noradrenaline 0.9 mcg/kg/min, and vasopressin 6 IU/L (vasoactive score 194). The arterial blood lactate was 12.4 mmol/L and the arterial pH was 7.25, following which the patient was classified as SCAI stage E. She was transferred to the operating room for immediate tMCS initiation. According to the local protocol, patients with cardiogenic shock and severely increased lactate levels (> 8 mmol/L) are scheduled to undergo ECMELLA 2.0 [4]. Briefly, the skin incision and preparation of the right axillary artery were performed in the infraclavicular fossa medial to the pectoralis minor muscle without division of the muscle fibers. Heparin was then administered, the artery clamped, and a 10-mm Y-shaped graft (Gelsoft, Vascutec Ltd, Renfrewshire, Scotland, UK) was anastomosed end-to-side to the axillar artery. After that, the graft was tunneled and externalized a short distance through a separate incision. This approach enables insertion of the Impella device through one branch of the Y-shaped graft and of the arterial cannula of the va-ECLS device through the other branch. While preparing the arterial artery access, transesophageal echocardiography (TEE) was performed and a floating left ventricular thrombus was detected. Consequently, implantation of the Impella was aborted and IABP was implanted in line with the current recommendations [1] to provide decompression of the distended left ventricle with subsequent va-ECMO implantation.

**Fig. 1:**
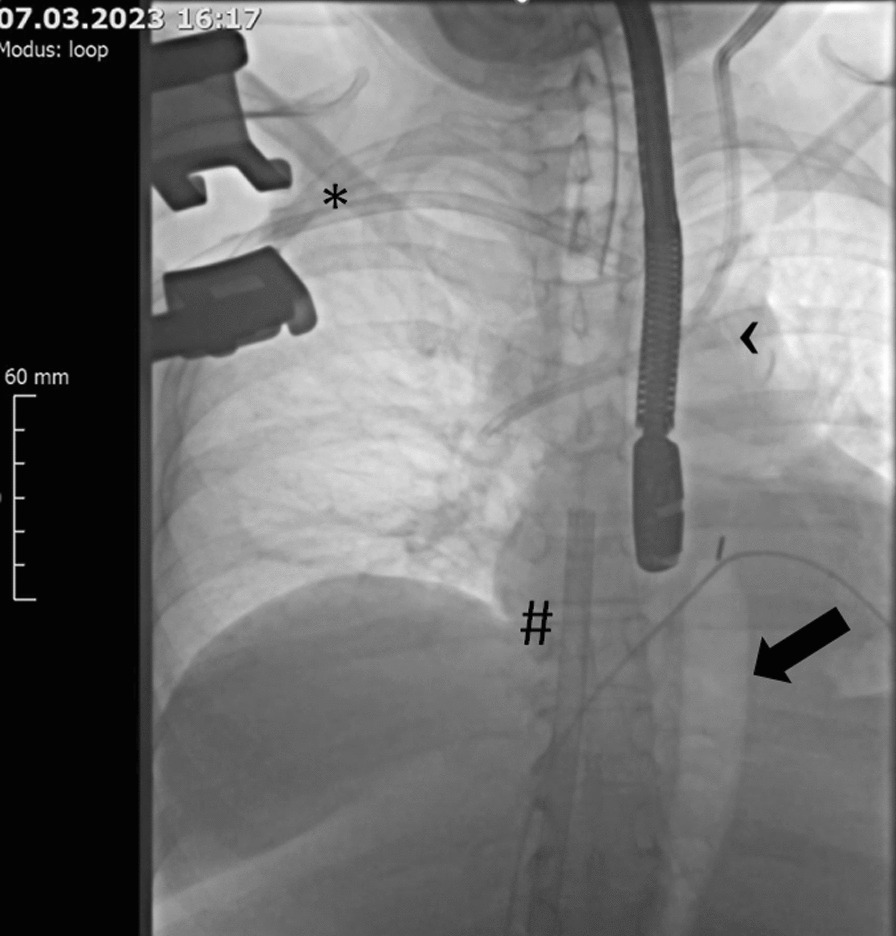
Chest X-ray showing veno-arterial extracorporeal life support and intra-aortic balloon pump simultaneously implanted via the right axillary artery. Arrow—inflated intra-aortic balloon pump. Arrowhead—intra-aortic balloon pump catheter leading to the right axillary artery. *Arterial cannula of the veno-arterial extracorporeal life support device implanted via the right axillary artery. # Venous cannula of the veno-arterial extracorporeal life support device implanted via the femoral vein

After IABP insertion via one branch of the Y-graft to the descending aorta below the left subclavian artery under fluoroscopic guidance, the patient suffered cardiac arrest and chest compression was initiated. The graft was ligated around the IABP introducer to address the diameter differences and the introducer was then secured to the lateral chest wall with multiple silk sutures. Alternatively, a hemostatic valve of the Impella device could be used to address diameter differences between the vascular graft and IABP shaft. A 17 Fr arterial cannula of va-ECMO was inserted via the second branch of the Y-graft. A 23 Fr venous cannula was inserted via the left femoral vein and advanced into the right atrium under TEE and fluoroscopic guidance, and both va-ECMO and IABP support were started (Fig. 1, Additional file 1: Video 1). Full ECLS flow was consequently achieved, however, despite all efforts, the patient passed away on the next day due to intractable multiple organ failure.

## Discussion and conclusions

According to observational studies, left ventricular unloading in patients supported with va-ECLS is feasible and associated with an improved survival [2]. Furthermore, early implantation of tMCS combined with efficient left ventricular unloading was associated with a mortality reduction and a higher likelihood of successful weaning from ventilation [5]. However, the Achilles heel of this approach remains a high burden of vascular complications including insertion site bleeding, insertion site ischemia requiring intervention, and lower limb ischemia distal to the device insertion site [2, 6]. Single-site insertion of both Impella and va-ECLS was previously proposed [3]; however, Impella insertion is contraindicated in the presence of a mechanical aortic valve, a mobile left ventricular thrombus, severe aortic stenosis, aortic dissection, and left ventricular outflow tract narrowing/obstruction [1]. In the majority of cases in which Impella insertion is contraindicated, IABP can be safely introduced as an alternative LV decompression device. Moreover, according to meta-analyses of retrospective studies, IABP is (albeit not unanimously) associated with a reduced mortality in patients treated with va-ECMO. Additionally, utilization of IABP is associated with a relatively low risk of complications and is considerably more affordable than an Impella device [7]. Additionally, antegrade arterial blood flow via transaxillary inserted ECLS may assist for better LV unloading compared to ECLS implantation via femoral cannulation that provides retrograde flow. However, these two approaches have not been compared in randomized studies.

**Fig. 2:**
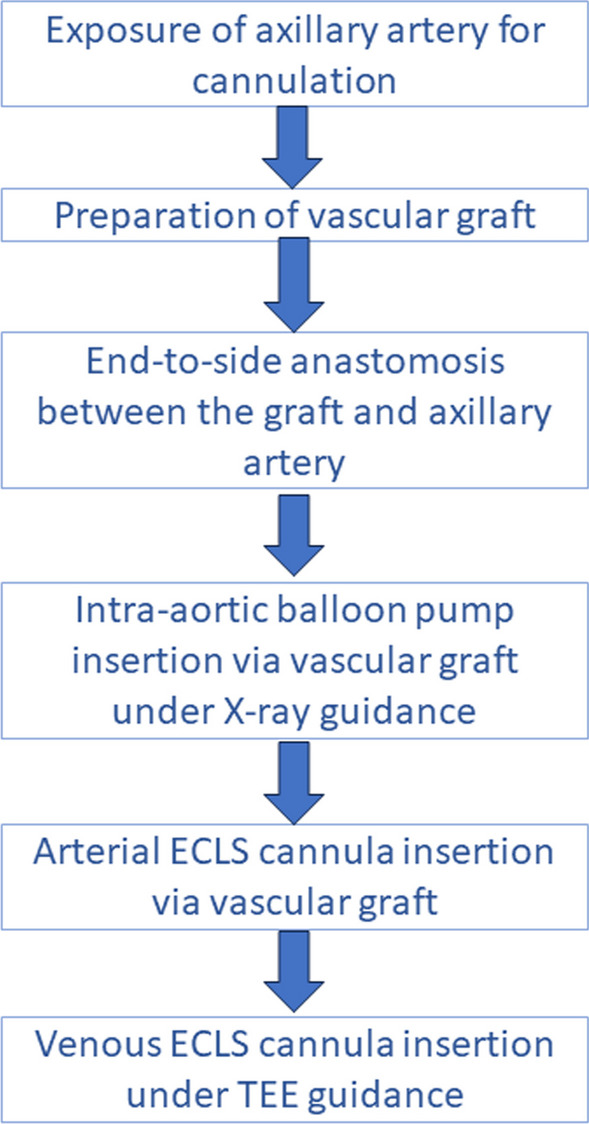
Summary of dual mechanical circulatory support insertion via single arterial site. *ECLS* extra corporeal life support; *TEE* transesophageal echocardiography

In this report we describe, to the best of our knowledge, the first implantation of va-ECLS and IABP using a single transaxillary arterial access site (Fig. 2). This concept might provide several advantages compared to the currently more common dual-site approach. First, the single-site technique might potentially reduce vascular complications associated with multidevice utilization. Second, transaxillary implantation eliminates the need for femoral cannulation, reduces the risk of groin infections, and makes distal perfusion placement redundant. The surgical application of an axillary graft was shown to be associated with a low risk of vascular injury and brachial plexus injury [8]. Third, this approach could be combined with jugular vein canulation for a venous va-ECLS cannula, thereby reducing the cannulation site for a single extremity and allowing early patient ambulation. Finally, utilization of a Y-graft enables a simple escalation from IABP to Impella at the bedside without the need for a surgical intervention or even an additional puncture. Furthermore, safe bedside weaning from va-ECLS is also possible under local anesthesia.

The major drawback of this technique is that time and expertise are needed to perform the surgical cutdown and vascular graft anastomosis. For this reason, this concept should be reserved for selected patients whose condition is stable enough for surgical dissection of the axillary artery.

In conclusion, implementation of this technique enables insertion of IABP and va-ECLS via a single arterial site, thereby diminishing the burden of potential vascular complications. Moreover, this concept might provide a simple and modular strategy for escalation of IABP to Impella and vice-versa, as well as weaning from tMCS in a simple bedside manner without the need for a separate arterial puncture.

### Supplementary Information

**Additional file 1. Veno-arterial extracorporeal life support and intra-aortic balloon pump.** This video describes VA-ECLS and IABP simultaneously inserted via single arterial access site.

## Data Availability

The data underlying this article will be shared on reasonable request to the corresponding author.
